# Contrasting distribution of enzyme activities in the rhizosphere of European beech and Norway spruce

**DOI:** 10.3389/fpls.2022.987112

**Published:** 2022-11-16

**Authors:** Bin Song, Bahar S. Razavi, Rodica Pena

**Affiliations:** ^1^ School of Geography and Ocean Science, Nanjing University, Nanjing, China; ^2^ Forest Botany and Tree Physiology, University of Göttingen, Göttingen, Germany; ^3^ Department of Soil and Plant Microbiome, Institute of Phytopathology, University of Kiel, Kiel, Germany; ^4^ Department of Agriculture Soil Science, University of Göttingen, Göttingen, Germany; ^5^ Department of Sustainable Land Management, School of Agriculture, Policy and Development, University of Reading, Reading, United Kingdom

**Keywords:** root morphology, traits, rhizoplane, zymography, temperate forests, gamma-irradiated soil

## Abstract

Recent policies and silvicultural management call for forest regeneration that involve the selection of tree species able to cope with low soil nutrient availability in forest ecosystems. Understanding the impact of different tree species on the rhizosphere processes (e.g., enzyme activities) involved in nutrient mobilisation is critical in selecting suitable species to adapt forests to environmental change. Here, we visualised and investigated the rhizosphere distribution of enzyme activities (cellobiohydrolase, leucine-aminopeptidase, and acid phosphomonoesterase) using zymography. We related the distribution of enzyme activities to the seedling root morphological traits of European beech (*Fagus sylvatica*) and Norway spruce (*Picea abies*), the two most cultivated temperate tree species that employ contrasting strategies in soil nutrient acquisition. We found that spruce showed a higher morphological heterogeneity along the roots than beech, resulting in a more robust relationship between rhizoplane-associated enzyme activities and the longitudinal distance from the root apex. The rhizoplane enzyme activities decreased in spruce and increased in beech with the distance from the root apex over a power-law equation. Spruce revealed broader rhizosphere extents of all three enzymes, but only acid phosphomonoesterase activity was higher compared with beech. This latter result was determined by a larger root system found in beech compared with spruce that enhanced cellobiohydrolase and leucine-aminopeptidase activities. The root hair zone and hair lengths were significant variables determining the distribution of enzyme activities in the rhizosphere. Our findings indicate that spruce has a more substantial influence on rhizosphere enzyme production and diffusion than beech, enabling spruce to better mobilise nutrients from organic sources in heterogeneous forest soils.

## Introduction

In forest ecosystems, anthropogenic pollution and climate change have resulted in nutrient limitation for trees (Jonard et al., 2015; Greaver et al., 2016; Marchi et al., 2018; Xue et al., 2022). This is particularly critical to seedling survival and growth in forest regeneration (Baier et al., 2006; Collet and Le Moguedec, 2007; Wagner et al., 2010). Forestry considers specific management practices (e.g., selective felling) to increase the benefic influence of mother trees on seedling nutrient acquisition through mycorrhizal mycelial networks (Figueiredo et al., 2021). However, empirical evidence suggests that mycorrhizal networking does not necessarily contribute to the seedling establishment and their nutritional improvement (Högberg and Högberg, 2022). In the absence of mature trees (e.g., regeneration in clear felling), seedlings perform better than in the presence of conspecific trees (Högberg and Högberg, 2022). One of the main mechanisms trees employ to alleviate nutrient limitation is the formation of a nutritional symbiosis with mycorrhizal fungi. The trees supply the fungi with photosynthetically fixed carbon in exchange for enhanced nutrient acquisition (Smith and Read, 2010). Young seedlings struggle to associate with mycorrhizal fungi in the first months of growth until the first flush of leaves becomes mature (Harley & Waid, 1955; Warren Wilson and Harley, 1983), and later, during the early stages, seedlings have a meagre mycorrhization rate (Harley, 1939; Warren Wilson and Harley, 1983; Newton and Pigott, 1991; Zeleznik et al., 2007; Pena et al., 2013; Nahberger et al., 2021). Therefore, the young seedlings, which are the most susceptible to nutrient deficiency among all other stages in the life of a tree, in the absence of mycorrhizal partners, must develop different strategies to overcome the nutrient limitation. One of these strategies is to intensify the rhizosphere processes, particularly the enzyme activities, which catalyze the nutrient release from soil organic matter (Marschner et al., 2011; Dijkstra et al., 2013; AminiTabrizi et al., 2022). The rhizosphere was broadly defined as the volume of soil influenced by root activity (Hiltner, 1904; Hinsinger, 1998). The term has been refined to comprise the endo- and ecto-rhizosphere, which consists of the rhizoplane (i.e., the root surface) and rhizosphere soil (Reinhold-Hurek et al., 2015; York et al., 2016). The ecto-rhizosphere is a hotspot of microbial activities and soil organic matter decomposition sustained by plant rhizodeposition (Ge et al., 2017; Macia-Vicente et al., 2020).

The plant influences the rhizosphere in vertical and horizontal directions (Luster et al., 2009; Minz et al., 2013; Wen et al., 2022). The roots are composed of segments that differ in their degree of development and differentiation showing specific physiological and biochemical characteristics: root cap, root tip, elongation zone, root-hair zone, mature zone, and sites of lateral root emergence. This root functional heterogeneity controls the root spatial abilities to take up nutrients (Luster et al., 2009; Hinsinger et al., 2011; Helliwell et al., 2017; Wang et al., 2022a). However, its effects on the spatial distribution of enzyme activities in the rhizosphere have been rarely investigated.

In the rhizosphere, the enzyme activities are determined by synergistic effects of plant and root-associated or soil microorganisms (Hinsinger et al., 2006; Kuzyakov and Razavi, 2019; Ren et al., 2021). The microorganisms rely on rhizodeposits (exudates, mucilage, or border cell loss, McCully, 1999; Brzostek et al., 2013; Zwetsloot et al., 2018) as easily accessible carbon (C) sources (Kuzyakov et al., 2000; Kuzyakov and Cheng, 2001; Huang et al., 2019; Herre et al., 2022). Thus, the plants contribute to the rhizosphere enzyme activities either directly by releasing enzymes or indirectly by influencing microbial abundance or activity through variation in the quantity and quality of rhizodeposition (Haichar et al., 2008; Pei et al., 2016; Uroz et al., 2016a; Zwetsloot et al., 2018). Furthermore, as the rhizoplane acts as a “regulatory gate” of microbial entry to the endosphere (van der Heijden and Schlaeppi, 2015), the plants also involve immunity and signalling mechanisms in controlling microbial distribution on the rhizoplane (Dupuy and Silk, 2016; Schmidt et al., 2018).

The rhizosphere enzyme activities are commonly interpreted as a response of plants and microorganisms to nutrient demand (Burns et al., 2013; Kang et al., 2022). The spatial distribution of enzyme activities in the rhizosphere may contribute to or reflect the plant ability to cope with nutrient limitations through better exploitation of soil resources (Hinsinger et al., 2011; Hummel et al., 2021). A comprehensive understanding of enzyme activities in the rhizosphere in relation to root morphological traits is a valuable contribution to selecting more adapted tree species to cope with nutrient limitations at young seedling stages.

European beech (*Fagus sylvatica* L.) and Norway spruce (*Picea abies* Karst) are the dominant species in temperate forest ecosystems in Central Europe (FAO, 2010; Leuschner and Ellenberg, 2017). They differ in terms of the chemical properties of the rhizosphere, root morphology and architecture, and the strategy by which they enhance nutrient acquisition (Wang et al., 2001; Calvaruso et al., 2014; Kolstad et al., 2016; Odriozola et al., 2020). Norway spruce has a shallow root system that commonly proliferates in the uppermost, organic-rich, soil layers, which contrasts with the beech tend toward a heart-shaped root system that branches out in both the upper and deeper soil layers (Schmid, 2002). In response to resource limitation, beech has a high level of plasticity in root biomass partitioning, a strategy not pursued by spruce, which has limited root plasticity (Matjaž and Primož, 2010; Schall et al., 2012). Nevertheless, spruce has a higher nutrient requirement and, consequently, higher nutrient depletion in the rhizosphere than beech (Wang et al., 2001).

In this study, we aimed to investigate the spatial distribution of enzyme activities on the rhizoplane and the rhizosphere enzyme activity extents (i.e., the horizontal distance from the root centre to bulk soil at which the enzyme activity decreases to a constant value) in relation with root morphological traits in European beech and Norway spruce young seedlings.

Rhizosphere enzyme activities change with plant nutrient requirements and physiology (Marschner et al., 2011), root morphology (Razavi et al., 2016; Giles et al., 2018; Ma et al., 2018a; Ma et al., 2018b), or rhizosphere microbial activity (Steinauer et al., 2016; Zhang et al., 2019; Kante et al., 2021). All these factors vary with plant species, life history or phylogeny (Garcıía et al., 2004; Minz et al., 2013; Brtnicky et al., 2021; Uroz et al., 2016b). Therefore, we hypothesised that (1) spatial distribution of enzyme activities in the rhizoplane is related to root morphological traits, and (2) Norway spruce show higher rhizosphere enzyme activities and broader rhizosphere extents than European beech. In this way, the spruce may complement its strategy of gaining access to organically bound nutrients at the expense of investment in root growth in deeper soil horizons that is the case with beech.

To test these hypotheses, we used a microcosm experiment where European beech and Norway spruce seedlings were planted in the soil, where fungi were absent. We used a γ-irradiated forest soil amended with soil-solution bacteria. In this way, we simulate the natural seedling establishment in the absence of ectomycorrhizal fungi that may influence rhizosphere enzyme activities (Firsching & Claassen, 1996).

To evaluate the enzyme activities and their spatial distribution in the rhizosphere, we employed zymography. For the investigation, we selected three enzymes, which are common in the temperate forest soil (Baldrian and Štursová, 2011), catalyse reactions of C, N, or P cycles and partially enabled us to disentangle the contribution of plant and microorganisms to the activity distribution pattern: cellobiohydrolase (CBH), which is mainly secreted by microorganisms to degrade cellulose into soluble sugars (Payne et al., 2015; Sanaullah et al., 2016); leucine-aminopeptidase (LAP), which targets proteinaceous compounds to release amino acids; and acid phosphomonoesterase (AP), which hydrolyses organic P-compounds to phosphates. Leucine-aminopeptidase and AP are secreted by both plants and microorganisms (Turner and Haygarth, 2005; Nannipieri et al., 2011).

## Material and methods

### Experimental setup

European beech (*Fagus sylvatica* L) and Norway spruce (*Picea abies* L. Karst) seedlings were grown in rhizoboxes for three months until the first flush of leaves was mature. This is also the stage when the root system is well-developed and considered fully developed for the season (Harley, 1940; Wilcox, 1968; Warren Wilson and Harley, 1983). The plants were obtained from seeds (Niedersächsische Landesforsten, Forstamt Oerrel, Germany), which were germinated on moist filter paper at 4°C in darkness for one week. When the radicles reached a length of 1-2 cm, the seedlings were sterilised following the procedure described by Pena et al. (2013). In short, after removal of the seed coats, the seedlings were immersed in a water solution of 0.1% fungicide and 0.1% tetracycline for 24 h (Proplant, Arysta LifeScience, Düsseldorf, Germany) (Duchefa Biochemie, Haarlem, Holland). The seedlings were thoroughly rinsed and then transferred immediately to rhizoboxes, where they were planted at a depth of 5 cm.

The rhizoboxes had inner dimensions of 21.5 × 11.4 × 3.6 cm and could be easily opened from the front. They were filled with sieved soil (mesh size 1000μm) to a bulk density of 1.5 g cm^-3^. This soil was loamy Haplic Luvisol, obtained by collecting the Ah horizon to a depth of 20 cm from a mature beech stand in the Hainich forest in Central Germany (51°04’46’’N, 10°27’08’’E). The soil contained 58 g kg^-1^ sand and 301 g kg^-1^ clay, with 36 g kg^-1^ of organic C and 3 g kg^-1^ total N, at a pH of 5.0 (Solly et al., 2014).

The soil was sterilised by γ-irradiation on two occasions at 30 kGy with a one-week interval between treatments (BGS Beta-Gamma-Service GmbH & Co, Wiehl, Germany). To minimise residual enzyme activities, the soil was kept in tightly closed containers at 4°C for one year (Lensi et al., 1991). Nevertheless, the bias of abiontic enzymes stabilised in the soil matrix (Nannipieri et al., 2018) was low as we used the same soil in all treatments.

The soil fungal contamination was monitored by spreading a 1.0 ml fresh soil solution on a Petri dish containing modifed Melin-Norkrans (MMN) agar medium and incubating the plates in darkness at 18°C for three weeks. Slight bacterial growth did occur, but no fungal growth was observed.

Before planting the soil, the rhizoboxes were amended with a bacterial culture stemming from a soil solution from the same forest site. The bacterial culture preparation was conducted according to the procedure described by (Nguyen et al., 2017). Each rhizobox was inoculated with 1.0 ml of bacterial solution diluted with 25 ml H_2_O.

After planting, the rhizoboxes (n=4) were transferred to a climate chamber maintained at a constant temperature of 22 ± 1°C, humidity 60%, and a daily light period of 14 h with an active photosynthetic radiation intensity of 250 μmol m^−2^ s^−1^. The plants were watered 2x/week with approximately 50 ml H2O per rhizobox. During the growth period, these boxes were inclined at an angle of 50° to facilitate root growth along their fronts.

### Soil zymography

**Figure 1:**
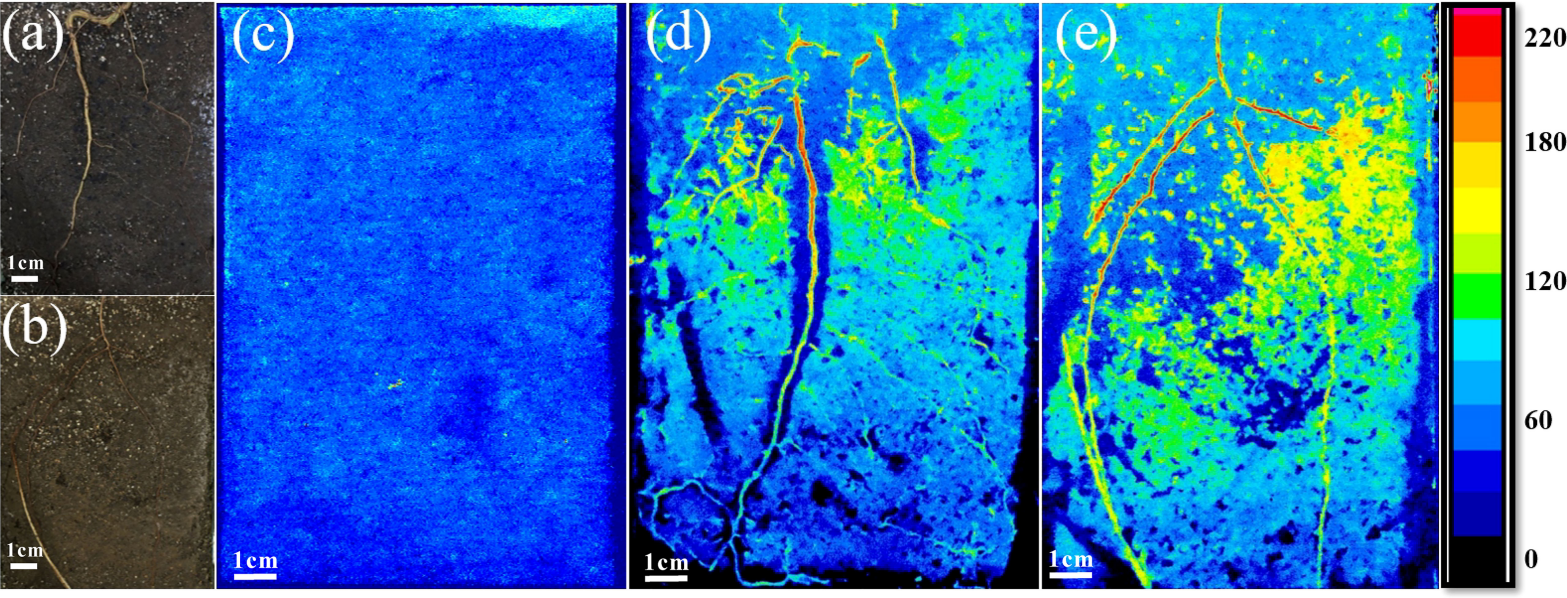
Roots of seedlings of European beech **(A)** and Norway spruce **(B)** grown in the rhizoboxes. Examples of zymographs showing the spatial distribution of acid phosphomonoesterase (AP) activity before planting **(C)**, and in the rhizosphere of European beech **(D)** and Norway spruce **(E)**. Side colour maps are proportional to the enzyme activities (pmol mm^−2^ h^−1^).

The spatial distribution of enzyme activities on the rhizoplane and in the rhizosphere soil was determined *in situ* using direct soil zymography before planting and when the plants were three-months-old (**Figure 1**). Soil zymography was conducted following the protocol from Razavi et al. (2019), with the measurements of cellobiohydrolase (CBH) (EC 3.2.1.91); leucine-aminopeptidase (LAP) (EC 3.4.11.1); and acid phosphomonoesterase (AP) (EC 3.1.3.2) activities. The applied method is based on the visualisation of enzyme activities using hydrophilic polyamide membranes saturated with either 4-methylumbelliferone (MUF)-substrates (pH 6.5) or 7-amino-4-methylcoumarin (AMC)-substrates (pH 7.2). The substrates become fluorescent when hydrolysed by the specific enzyme (Dong et al., 2007). The membranes (Tao Yuan, China) were 100 µm thick with a pore size of 0.45 µm. Cellobiohydrolase activity was detected using 4-methylumbelliferyl-β-D-cellobioside (MUF-C), LAP activity by L-leucine-7-amino-4-methylcoumarin hydrochloride (AMC-L), and AP activity by 4-methylumbelliferyl-phosphate (MUF-P) substrate. MUF substrates were dissolved separately to a concentration of 12 mM in an MES buffer (C_6_H_13_NO_4_SNa0.5, Sigma-Aldrich, Darmstadt, Germany). AMC substrate was dissolved in TRIZMA (C_4_H_11_NO_3_ ·HCl, C4H_11_NO_3_, Sigma-Aldrich, Darmstadt, Germany). The rhizoboxes were carefully opened, and the membrane, previously saturated with the enzyme-specific substrate, was applied directly to the root surface. For each rhizobox, a soil zymography test was performed separately for each enzyme in the following order: CBH, AP, and LAP (Ma, Liu, et al., 2018). Each membrane was incubated on the soil surface for one hour and then gently lifted off with tweezers. After incubation, the membranes were placed on a transparent laser imaging cover (35 × 43 cm, Carestream, NY, USA), transferred to the darkroom, and photographed under ultraviolet (UV) illumination at an excitation wavelength of 355 nm and an emission wavelength of 460 nm. The camera was a Sony DSC-RX100m5 (Sony, Tokyo, Japan) with 35 mm focal length and 20 megapixels, mounted on a Rocwing Pro Copy Stand (Rocwing Co, London, UK). To ensure uniform illumination of the membranes, three 22W purple fluorescent lamps (Damar Worldwide 4 LLC, Aurora, U.S.A.) were fitted to the camera as a source of UV light. For all measurements, the distance, aperture, and shutter speed of the camera were set to 250 mm, f/5.0 and 1/250 sec, respectively.

### Calibration and validation of soil zymograms

To analyse and quantify the zymogram pictures, a standard calibration curve was plotted relating the enzyme activity to the gray-value of zymogram fluorescence using serial dilutions of MUF (0, 0.01, 0.05, 0.1, 0.2, 0.4, 0.6, 0.8, and 1.0 mM) and AMC (0, 0.01, 0.1, 0.2, 0.5, 1.0, 2.0, 3.0, 4.0, 5.0, and 6.0 mM). The membranes were cut into 4 cm^2^ pieces and soaked in these solutions for one hour. The amount of MUF and AMC on an area basis was calculated based on the size of the membrane and the volume of solution taken up (Guber et al., 2018). The membranes were placed in the transparent laser imaging cover and photographed in UV light at the camera setting described above. These calibration membranes were imaged under UV light following the same procedure as with the rhizobox samples.

The zymogram quantification was conducted with a Matlab toolbox (MathWorks, Natick, Massachusetts, USA). The fluorescence spots on the zymogram images show the areas in which the substrate was enzymatically hydrolysed. The intensity of fluorescence is proportional to the enzyme activity. To calculate the enzyme activity, the clearly visible, not overlapping roots were selected and subsequently, skeletonised and plotted using the Thinning and Image acquisition (Euclidean distance map) functions (Zarebanadkouki et al., 2014). Images were transformed to 16-bit grayscale images as matrices and corrected for light variations and camera noise (Razavi et al., 2016; Wei et al., 2019a). The total activity of every single enzyme in the rhizosphere was calculated by assessing the sum total of the pixel values in the region between the root centre and border of the rhizosphere. The grey-value from the blank region of samples was set as the background, and, subsequently, subtracted from the total pixel value. To calculate the enzyme activities, the *Analysis* function of ImageJ Fiji (National Institutes of Health, USA) was employed. On the rhizoplane, the measurements included 340 points up to a longitudinal distance of 7.0 cm from the root tip. In the rhizosphere soil, the enzyme activities were measured laterally from the root centre at seven points, equally distributed over 1.8 mm. The lateral measurements were conducted on seven vertical levels, and subsequently, the mean value for each horizontal point was derived. The standard calibration curve was used to convert the all grey-values from the various images to enzyme activities using STATISTICA software (Dell, Texas, USA) according to the procedure described by Guber et al. (2018) and Razavi et al. (2019).

The enzyme activities were measured on the rhizoplane and in the rhizosphere soil laterally from the taproot in beech and all (three to five) main roots in spruce. In the latter case, the mean values were calculated and used in the analysis. The reason for using all main roots in spruce was the formation of horizontal side roots of similar size to the taproot that is a characteristic of spruce seedlings (Puhe, 2003). In young beech seedlings, on the contrary, the taproot comprises the main part of the root system, with the lateral roots becoming functionally important only later in the ontogeny (Harley, 1939; Harley, 1940). The visual assessment of zymograms confirmed that the most intense enzyme activities occurred on the tap root in beech and all main (tap and side) roots in spruce (**Figure 1**).

### Sampling and analysis of root morphology

Following the zymography measurements, the plants were harvested and separated into the shoot and root segments. The roots were carefully washed to remove all soil particles, briefly dried with paper tissues, and weighed to register the fresh biomass. After the morphological analysis, the roots were separated into fine and coarse roots, and aliquots of both categories were oven-dried at 60°C to constant weight; these were then used for assessing the dry biomass.

A root morphological analysis was conducted using WinRHIZO software (Regent Instruments Inc., Ottawa, Canada). The entire roots were evenly spread within a thin layer of tap water in a transparent tray and scanned at a resolution of 400 dpi using a flatbed scanner (ScanMaker i800pluS, Microtek, China). The images of these scanned root samples were saved in the TIF format and then imported to WinRHIZO software. The software was set up as suggested by Lobet et al. (2011) and Pierret et al. (2013): *Image mode* 8-bit Gray; *Resolution* 400 dpi; *Scale* 100%; *Calibration* intrinsic for scanner; *Root/background* and *Colour analysis* based on grey levels (threshold)—Manual—Dark on white background; *Particle* cleaning on. The WinRHIZO data output included total root length, main root length, surface area, mean diameter, root volume, number of tips, and number of forks. The 7 cm long roots, which were analysed for enzyme activities, were excised and placed in a Petri dish with tap water and photographed (Leica DFC 420 C; Leica, Wetzlar, Germany) under a stereomicroscope (Leica M205 FA). The extent of the root elongation zone (the area from the apex until the first root hairs) and root hair zone, root hair length, and root diameter were measured along the root fragments at the points where enzyme activities were measured using the tool MRI Root Hair Tools implemented in ImageJ (https://github.com/MontpellierRessourcesImagerie/imagej_macros_and_scripts/wiki/MRI_Root_Hair_Tools).

### Calculations and data analysis

The normalised enzyme activity was calculated by dividing the total enzyme activity by the root surface area, as described by Ma et al. (2018b).

The nutrition acquisition ratio in the rhizosphere was calculated as following: C/N acquisition = ln (CBH)/ln (LAP), N/P acquisition = ln (LAP)/ln (AP), and C/P acquisition = ln (CBH)/ln (AP) (Wei et al., 2019b; Karhu et al., 2022).

The enzyme activity (y), as a function of vertical distance from the root tip (x), was fitted by a power function (Niklas, 1994):


$$y=ax^{b}$$

where a is the allometric coefficient, and b is the regression coefficient or scaling exponent.

The enzyme activity (y) as a function of horizontal distance from the root centre (x) was fitted by applying the sigmoid Hill equation:


$$y=min+\frac{\left(max-min\right)}{1+{\left(x/EC_{50}\right)}^{-Hill\;slope}}$$

The criteria of equation choice were the best description of the observed pattern of data distribution. The curve fitting was conducted with Origin (Pro) software (OriginLab Corporation, Northampton, MA, USA).

The differences between the plant species concerning the root characteristics, enzyme activities and scaling exponents of fitted curves (Sokal and Rohlf, 2013) were assessed using Student’s t-test or one-factor ANOVA; p-values< 0.05 were used to indicate significant differences between the means. Levene’s and Shapiro–Wilk’s tests were used to check for data homogeneity and normal distribution. Principal Components Analysis (PCA) was employed to evaluate the relationships between root morphological characteristics and enzyme activities. The analyses were conducted with R 3.6.0 statistical software using the following functions: levene.test() and qqPlot(), car-package (Fox and Weisberg, 2018); shapiro.test(), aov(), TukeyHSD(), t-test(), stats-package; and princomp(), vegan-package (R Core Team, 2020).

## Results

### Variation in root morphology of European beech and Norway spruce

Total root length and surface area were four and ten times larger in beech than in spruce (**Table 1**). The number of root tips and biomass were also 10 times higher in beech than in spruce (**Table 1**).

**Table 1:** Root characteristics of young beech and spruce seedling.

	Beech	Spruce
Total length (cm)	1196 ± 47 b	216.4 ± 33.1 a
Taproot length (cm)	19.55 ± 0.98 b	13.87 ± 1.13 a
Surface area (cm^-2^)	189.9 ± 7.8 b	28.73 ± 4.51 a
Mean Diameter (mm)	0.51 ± 0.02 b	0.40 ± 0.01 a
Volume (cm^-3^)	2.64 ± 0.08 b	0.28 ± 0.06 a
Tips	4452 ± 207 b	732 ± 75.3 a
Forks	6775 ± 487 b	689 ± 51 a
Root hair length (mm)*	0.06 ± 0.01 a	0.32 ± 0.03 b
Biomass (g)	3.32 ± 0.29 b	0.41 ± 0.01 a

*Values obtained by stereomicroscope image analysis. Values represent the mean ( ± SD). Different letters indicate significant statistical differences between European beech and Norway spruce obtained by Student’s t-test, p < 0.05. N = 4.

In beech, the mean root diameter was 20% larger than in spruce (**Table 1**), and the taproot increased with the distance from the root apex (**Table 2**). In spruce, no relationship between root diameter and the distance from root apex was apparent (**Table 2**).

**Table 2:** Correlation matrix (Spearman r) of root hair length and root diameter with the longitudinal distance from the root apex.

		Root hair length	Diameter
Distance from the root tip			
Beech		0.010	**0.180**
Spruce		**0.400**	0.020

Bolded numbers indicate a significant relationship between European beech and Norway spruce, p < 0.05. (N = 340).

**Figure 2:**
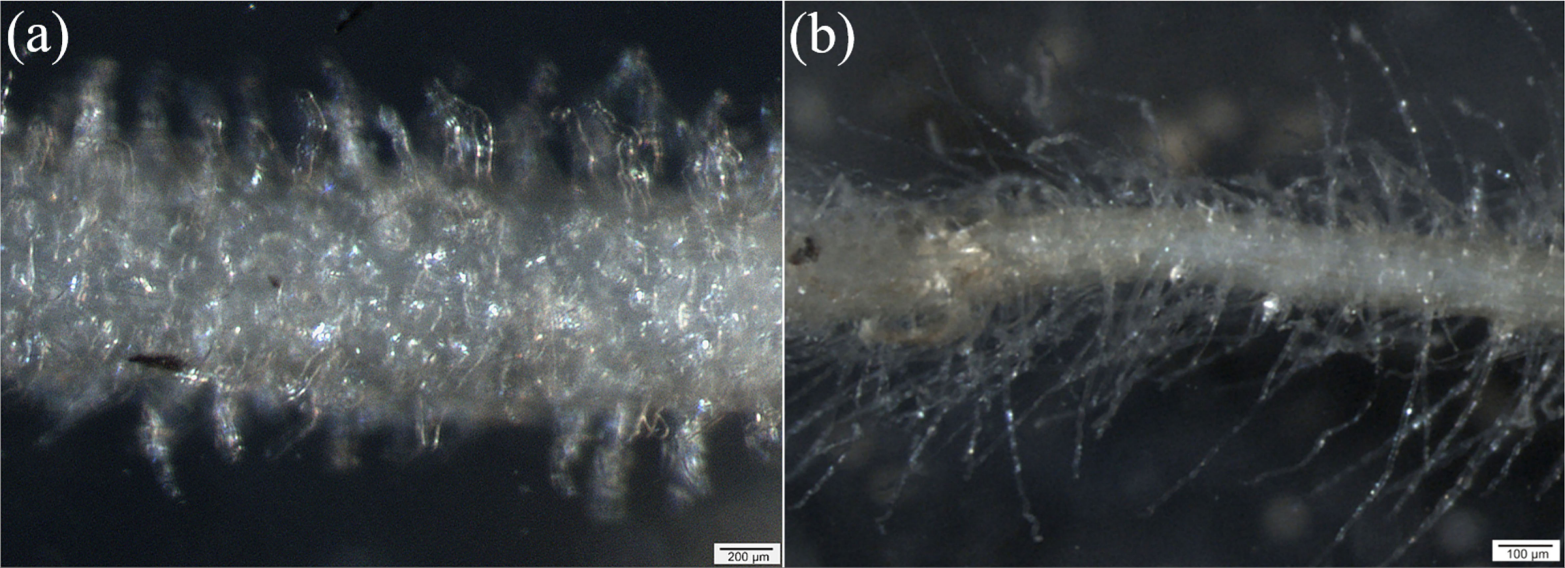
Stereomicroscope images of root hair zone of European beech **(A)** and Norway spruce **(B)**.

The root hair zone started at 0.13 ± 0.005 mm and 0.33 ± 0.021 mm distance from the root apex in beech and spruce, respectively. In beech, the root hair length (RHL) was in the range of 0.03 to 0.04 mm (**Figure 2**). A relatively small percentage (2.4%) of root hairs reached longer lengths. Root hair length was unrelated to the longitudinal distance from the root apex (**Table 2**). In spruce, RHL was more heterogeneous, ranging from 0.2 to 0.5 although about 70% of the root hairs showed an RHL ranging from 0.3 to 0.5 mm (**Figure 2**). Root hair length significantly increased with the longitudinal distance from the root apex (**Table 2**). Thus, various root characteristics of beech (e.g., length, surface area, diameter, and biomass) were superior to those of spruce, even though spruce had overall longer root hairs than beech (**Figure 2**).

### The longitudinal distribution of enzyme activities on the rhizoplane

**Figure 3:**
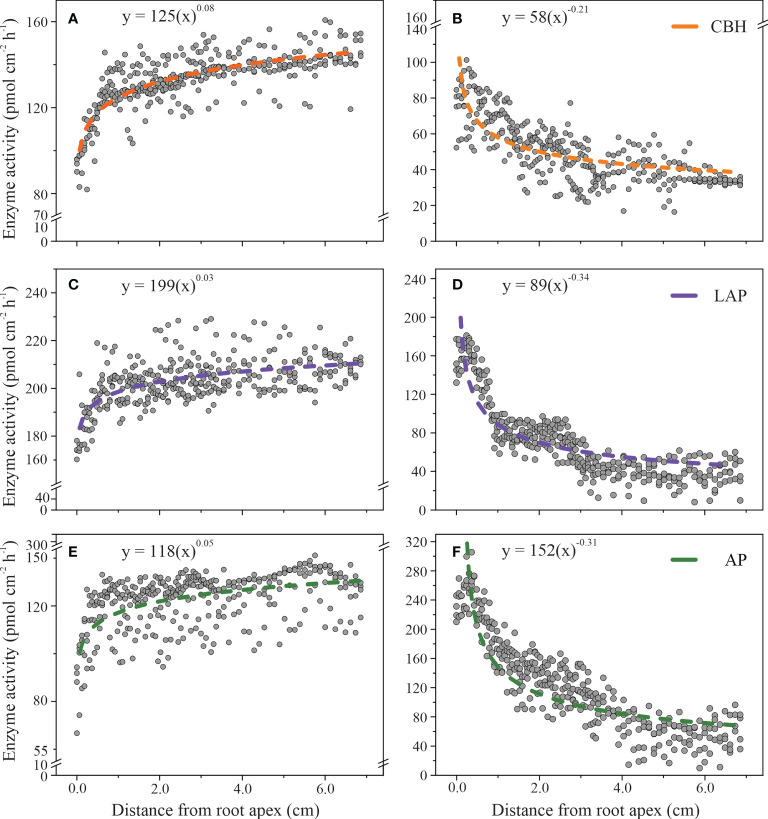
Enzyme activities on the rhizoplane as a function of the longitudinal distance from the root apex to the distal root zone in European beech **(A, C, E)** and Norway spruce **(B, D, F)**: cellobiohydrolase (CBH), leucine-aminopeptidase (LAP), and acid phosphomonoesterase (AP). The models include data from 85 individual measuring points per plant. (N = 4).

The spatial distributions of enzyme activities on the rhizoplane in relationship with the distance from the root apex were described by a power-law in beech and an inverse power-law equation in spruce (**Figure 3**). The order of scaling was about ten times higher in spruce than in beech (**Figure 3**), indicating a larger difference in enzyme activities along the roots in spruce than in beech. The largest difference in the scaling order between beech (0.03 ± 0.002) and spruce (0.34 ± 0.01) was in LAP activity. In beech, LAP activity slightly increased from 180 ± 3 (pmol cm^-2^h^-1^) at the root apex to 210 ± 1 (pmol cm^-2^h^-1^) at 7.0 cm from the root tip (**Figure 3C**), while in spruce it decreased from 188 ± 5 (pmol cm^-2^h^-1^) to 27 ± 4 (pmol cm^-2^h^-1^) at the same distance from the root apex (**Figure 3D**). The trend was similar for CBH activities (scaling order, 0.08 ± 0.003 in beech; 0.21 ± 0.009 in spruce, **Figures 3A, B**) and AP (0.05 ± 0.004 in beech; 0.31 ± 0.010 in spruce, **Figures 3E, F**).

The patterns of the spatial distribution of enzyme activities along the root growth axis were associated with the increase in root diameter in beech, and a decrease in root hair length in spruce, as the two parameters were correlated with the distance from the root apex (**Table 2**). To investigate whether this association with root parameters was independent of tree species, we evaluated the association of root hair length in beech (**Figure S1**) and root diameter size in spruce (**Figure S2**) with distance from the root apex in distinct root zones, characterised by specific classes of root hair lengths or root diameters, respectively. In beech, in the root areas in which no root hairs occurred, - primarily at the root apex, the power-law equation did not adequately describe the spatial relationship concerning any of the measured enzyme activities (SE = 0.120 for CBH; SE = 0.140 for LAP; SE = 0.226 for AP, **Figure S1**). Similarly, in the root areas where root hair length exceeded 0.04 mm, the power-law function failed to fit the data (SE = 0.047 for CBH; SE = 0.030 for LAP; SE = 0.119 for AP, **Figure S1**). The root areas of root hair lengths of 0.3 and 0.4 mm, representing the largest rhizoplane area, revealed a close relationship between enzyme activities and the distance from the root (**Figure S1**). This relationship was particularly prominent in CBH activity, which increased with the distance from root apex both in the 0.3 mm-root hair length (0.08 ± 0.005) and 0.4 mm- root hair length root areas (0.07 ± 0.002, **Figure S1**).

In spruce, in areas of the smallest (≤ 0.24 mm) and largest (≥ 0.28 mm) root diameter size (**Figure S2**), the distribution of enzyme activities poorly fitted a power-law equation. In the root areas where the diameter was 0.25 cm, 0.26 cm, and 0.27 cm, the enzyme activities followed the power-law relationship concerning the distances from the root apex (0.018 ≤ SE ≥ 0.038; **Figure S2**).

### The relationships between root morphology and enzyme activities in the rhizosphere soil

**Figure 4:**
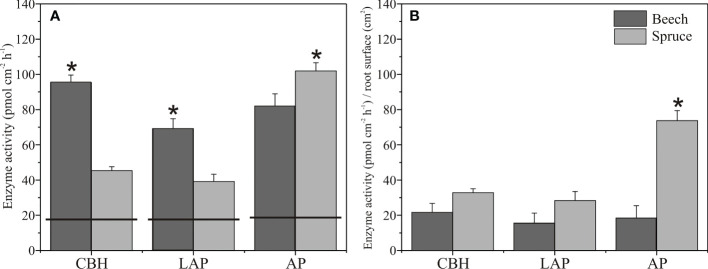
Enzyme activities in the rhizosphere of European beech and Norway spruce. Total **(A)** and root surface area-normalised **(B)** activity of cellobiohydrolase (CBH), leucine-aminopeptidase (LAP), and acid phosphomonoesterase (AP). The horizontal lines on the bars indicate the enzyme activities in the soil before tree planting. Bars represent mean values, and error bars represent standard error of the mean (N = 4). Asterisks indicate significant differences for means of enzyme activities between beech and spruce, *p*< 0.05.

Enzyme activities in the soil assessed from zymograms before planting were in a similar range of 18 to 19 pmol cm^-1^ h^-1^ for CBH and LAP, and AP, respectively (**Figures 1**, **4**). In the rhizosphere of three-month-old beech seedlings, CBH and LAP activities were about twice as high as in the spruce rhizosphere (**Figure 4A**). Acid phosphomonoesterase activity, on the contrary, was higher in the spruce than beech rhizosphere (**Figures 4A**, **1**). Carbon/Nitrogen acquisition ratio (ratio of C-acquiring to N-acquiring enzyme activities) was close to 1.0 and did not differ between beech and spruce (**Table S1**). Acquisition ratios of C/P and N/P were higher in beech than in spruce, with values<1.0 in the spruce (**Table S1**).

When the enzyme activity values were normalised to the root surface area, no differences occurred between the spruce and beech rhizosphere CBH and LAP activities (**Figure 4B**). However, AP activity was about four times higher in the spruce than in the beech rhizosphere (**Figure 4B**).

**Figure 5:**
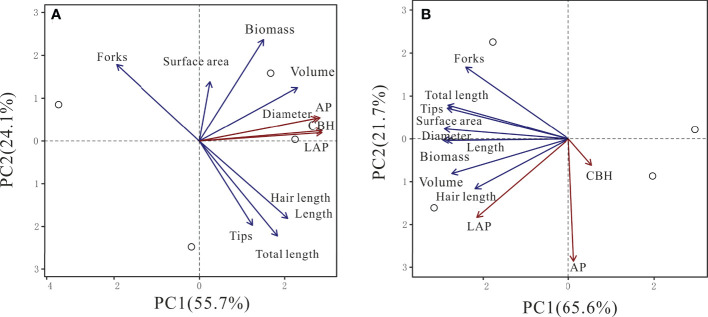
Principal component analysis (PCA) of cellobiohydrolase (CBH), leucine-aminopeptidase (LAP), and acid phosphomonoesterase (AP) activities in relationship with root traits in beech **(A)** and spruce **(B)** rhizosphere. (N = 4).

In beech, CBH, LAP, and AP activities were positively associated with the first principal component (PC1, 55.7%) together with root diameter and volume (**Table S2**, **Figure 5**). All other root parameters were also positively associated with PC1 but to a lower magnitude. In spruce, only LAP activity was associated with PC1 (65.7%), together with all root morphological traits (**Table S2**, **Figure 5**). The second PC (21.7%) had largely negative associations with LAP and AP activities, and root diameter (**Table S2**, **Figure 5**).

### The extents of enzyme activities in the rhizosphere soil

**Figure 6:**
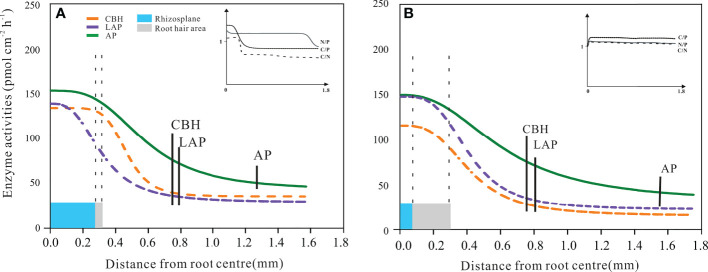
Distribution of enzyme activities in the European beech **(A)** and Norway spruce **(B)** rhizosphere. The curves represent the Hill function fitting the enzyme activity related to the horizontal distance from the root centre. Cellobiohydrolase (CBH), leucine-aminopeptidase (LAP), and acid phosphomonoesterase (AP). The vertical dotted lines represent the size of the root radius (blue area) and root hair length (grey area). The vertical solid bars represent the end of the extent of enzyme activity for CBH, LAP, and AP. The models include the mean data from 7 vertical measuring points per radial measurement point per plant. (N = 4). Error bars of enzyme activities were omitted to improve visualisation; the standard errors were always less than 10% of the activity values.

The profile of rhizosphere enzyme activities as a function of the outward distance from the root centre was described by the Hill equation. This took on different forms in the beech and spruce samples (**Figure 6**). For all measured enzymes – both in beech and spruce – the maximum value was apparent on the root surface and started to decrease at the root radius edge (**Figure 6**). An exception was the beech LAP activity, which gradually decreased from the root centre toward the end of the root radius (**Figure 6**).

The inflexion point of the CBH activity function from the root centre was 0.5 mm for beech and 0.4 mm for spruce (**Figure 6**). The rhizosphere extent of CBH activity differed (*P* = 0.038) between beech (0.72 ± 0.05 mm) and spruce (0.80 ± 0.04 mm). There was a more abrupt decrease in beech CBH activity (Hill coef. = 6.5) in response to the distance from the root centre as compared to spruce CBH activity (Hill coef. = 3.0 for spruce, **Figure 6**). The shape of the beech CBH activity curve indicated that the decrease in CBH activity started where the root hair area ended (**Figure 6A**). The influence of root hairs also became apparent through the linear correlation between the extent of RHL and rhizosphere CBH activity (**Table 3**).

**Table 3:** Pearson’s correlations between root hair lengths and rhizosphere extents of cellobiohydrolase (CBH), leucine aminopeptidase (LAP), and acid phosphomonoesterase (AP) in European beech and Norway spruce. N = 28.

	CBH	LAP	AP
Root hair length			
Beech	0.833	0.228	0.020
Spruce	0.690	0.320	0.010

Leucine-aminopeptidase activity in the beech rhizosphere continued until 0.78 ± 0.03 mm but with a gradual decrease (Hill coef. = 2.5) (**Figure 6A**). Likewise, in spruce, LAP activity was present until 0.84 ± 0.11 mm (Hill coef. = 3.6) (**Figure 6B**). In both species, LAP activity was not related to root hair lengths (**Table 3**).

Hill AP activity curve in the beech and spruce rhizospheres produced different shapes as compared to those of CBH and LAP (**Figures 6A, B**). The inflexion points of the curve from the root centre corresponded to 0.6 mm for beech and 0.7 mm for spruce (**Figure 6**). Acid phosphomonoesterase activity displayed the broadest rhizosphere distribution among the three enzyme activities, both in beech (distance from the root centre = 1.22 ± 0.07 mm) and spruce (1.58 ± 0.03 mm), with significant differences (p< 0.005) between the tree species (**Figure 6**). The curve shape indicated that there was no effect of the root hair size on AP activity (**Figures 6A, B**). There were no linear relationships between AP activity and the length of root hairs (**Table 3**).

In beech, C/P and C/N acquisition ratios decreased<1.0 with decreasing CBH activity at the edge of root hairs, while N/P ratio remained constant in the rhizosphere soil (**Figure 6A**, inset). In spruce, all acquisition ratios were maintained constant, close to 1.0 (**Figure 6B**, inset).

## Discussion

In this study, we investigated the spatial distribution of enzyme activities on the rhizoplane and in the rhizosphere soil of European beech and Norway spruce in relationship with root morphology. In accordance with previous studies (Bolte and Villanueva, 2006; Schall et al., 2012), we found that the size of the root system (i.e., root length, surface, mean diameter, biomass, and number of forks and root tips) was more extensive in beech than spruce. This finding confirms the beech nutrient uptake strategy to massively proliferate in exploiting a large soil volume (Leuschner et al., 2001).

The taproot zones, considered a proxy of longitudinal root heterogeneity, were more evident in spruce than in beech. The root apex, elongation zone, and root hair zones were broader in spruce than in beech. The root hair lengths presented a higher variation and were on average more than ten times greater in spruce than in beech.

### The enzyme activities on the rhizoplane increase with root diameter and decrease with root hair lengths

The results confirmed our first hypothesis that the spatial distribution of enzyme activities in the rhizoplane is related to root morphological traits. We found that the spatial distribution of enzyme activities along the root growth axis was associated with the increase in root diameter in beech, and a decrease in root hair length in spruce.

Distribution of CBH, LAP or AP activities on the rhizoplane in relation to the longitudinal distance from the root apex followed a power law and an inverse power law equation in beech and spruce, respectively. Razavi et al. (2016) have also reported differences in the distribution of enzyme activities along rhizoplane among herbaceous plants, but the contrast was less apparent than in the selected tree species. However, the order of scaling was relatively small in beech compared with spruce, suggesting a relative homogeneity in the spatial distribution of enzyme activities along the beech roots. This result was explained by the more homogeneous morphological appearance of beech roots compared with spruce. The root morphological and physiological heterogeneity results in the alteration of the chemical composition of exudates (Jaeger et al., 1999; Wen et al., 2022) or generate micro-niches, where the mucilage and other exudates may accumulate, favouring microbial activity (Massalha et al., 2017; Schmidt et al., 2018; Li et al., 2020; Xiong et al., 2021). As the enzymes cleave complex organic compounds from rhizodeposits into absorbable nutrients for plants and microorganisms (Nannipieri et al., 2007; Nannipieri et al., 2011), the quantity and quality of rhizodeposits are likely contributing to the spatial distribution of enzyme activities on the rhizoplane. A particularly important component of root heterogeneity is the root hair zone that prevails in comparison with other root zones in exchange fluxes with the environment, mainly nutrient uptake (Bidel et al., 2000; Laporte et al., 2013; Nguyen, 2003), or the release of exudates (Holz et al., 2018). The root hair zone supports the presence and wave-like distribution of microorganisms (Semenov et al., 1999; Zelenev et al., 2000). This explains our findings in beech roots of a stronger relationship between enzyme activities and distance from the root tip in the root hair zone than in other zones (**Figure S1**). Although we cannot disentangle the contribution of root or microbial activity to the rhizoplane enzyme activities, we posit that the root hair presence likely stimulated microbially- than plant-derived enzymes. We developed this proposition as in the root hair zone the microbe-specific CBH showed a more robust relationship than the dual (plant and microbially derived) AP activity.

In spruce, we found a decrease in enzyme activities from the root apex to the elongation zone and further to the root hair zone. The root hair lengths increased with the distance from the apex. The high enzymatic activity at the root apex has been previously described (Kuzyakov & Razavi, 2019) and possibly explained by a higher microbial enzymatic activity (Kuzyakov et al., 2000) triggered by the high amount of released exudates at the root apex (Watt et al., 2006; Hinsinger et al., 2009). However, we cannot exclude that the higher apical activities are due to a larger contribution of plant-released enzymes. Root apical parts are involved in taking up specific nutrients (Häussling et al., 1988) and exude enzymes more intensively than mature parts (Godlewski and Adamczyk, 2007). Moreover, plant and microbial uptake of nutrients (i.e., phosphorus) spatially differ along with the roots: the root apical part is reserved for the plant, while the root hair zone is for microbial P uptake (Marschner et al., 2011; Spohn and Kuzyakov, 2013). We speculate that the plant-released enzymes significantly contribute to the spatial distribution of enzyme activities on the rhizoplane. In support of this assumption, we found a lesser relationship between microbe-specific CBH activity and distance from the root tip than AP and LAP, which both plants and microorganisms can release.

### The rhizosphere CBH and LAP, but not AP activities were higher in beech than in spruce

The more extensive root system of beech triggered higher CBH and LAP activities in beech than in spruce rhizosphere. We obtained no differences in CBH and LAP activities between beech and spruce by considering the differences in root surface area. However, AP activity was higher in spruce than beech, regardless of the root size. This result was surprising because in beech, similarly to CBH and LAP, and in accordance with other studies (Meller et al., 2020), AP activity was largely positively associated with root morphological parameters, particularly root mean diameter and root volume. We may explain this discrepancy through the complexity of factors that control the rhizosphere AP activity (Nannipieri et al., 2011; Margalef et al., 2017; Nannipieri et al., 2018). The physiological status of the plant (Clausing et al., 2021), species or genotype identity (Denton et al., 2006; Razavi et al., 2016; Ma et al., 2018b; Meller et al., 2020), or soil phosphorus level (Hofmann et al., 2016; Wang et al., 2022b) contribute to AP activity in the rhizosphere.

The acid phosphatase was the only enzyme which supported the first part of our second hypothesis that enzyme activities are higher in spruce than in beech rhizosphere. Rejsek et al. (2012) have reported higher AP activity in spruce than beech forests because of differences in plant-released AP activity. In contrast, microbial-derived AP activity was similar in beech and spruce soil (Rejsek et al., 2012). Other studies also have suggested a high contribution of the plant over microbial AP activity in the rhizosphere (Nannipieri et al., 2011; Rejsek et al., 2012; Spohn & Kuzyakov, 2013; Hou et al., 2015).

Cellobiohydrolase and LAP activities were higher in beech than in spruce rhizosphere primarily because of the root size. Cellobiohydrolase CBH was related to root morphology in beech, but not in spruce, while LAP was the only enzyme related to root morphological parameters both in beech and spruce. In contrast with AP activity that may consist of a significant plant-derived contribution, CBH (Payne et al., 2015; Sanaullah et al., 2016) and LAP (Zhang et al., 2019; Greenfield et al., 2020) activities in the rhizosphere are likely produced by microbial enzymes. Thus, CBH and LAP activities were related to the root size as microbial activity relies on root exudation (Kuzyakov and Razavi, 2019) that positively correlates with the root size (Badri and Vivanco, 2009; Kuzyakov and Razavi, 2019) and root morphology (Meier et al., 2020). Moreover, CBH is involved in plant cell degradation by microorganism’s penetration in the endosphere (Schmidt et al., 2018) and the degradation of dead root fragments (Berlemont and Martiny, 2013; López-Mondéjar et al., 2016). A larger root surface and amount of dead material that increases with the root size may contribute to the observed higher CBH activity in beech than in the spruce rhizosphere.

The nutrient acquisition ratio representing the microbial investment in acquiring nutrients to maintain their internal stoichiometry for C/N was similar between beech and spruce and close to 1.0, indicating no microbial C vs N limitations (Sinsabaugh and Shah, 2012). The acquisition ratios of C/P and N/P were< 1.0 and higher in spruce than in beech. However, we cannot interpret them in the way of P limitation, although a higher AP activity is commonly related to P depletion (Nannipieri et al., 2011), as AP activity was related to trees and not related to microorganism requirements.

### Broader AP and CBH activity extents in spruce than beech rhizosphere soil

The extents of enzyme activities in the rhizosphere were in the order AP > LAP > CBH in both species. This finding is consistent with those of Razavi et al. (2016) and Ma et al. (2018b). They reported the same pattern in herbaceous plants, likely reflecting the plant and microbial demand for nutrients or, in the case of microorganisms, the availability of rhizodeposits, which represent their primary energy source (Burns et al., 2013).

The rhizosphere extents of AP activity were more significant in spruce than beech, supporting the higher AP activity found in the spruce rhizosphere. Taken together, these results indicate a higher potential to mobilise P from organic sources for spruce than beech. We speculate that better access to organic P reflects the spruce strategy to cope with nutrient limitation at the expense of investment in root development (Matjaž and Primož, 2010; Schall et al., 2012). Nevertheless, caution is due here as the measured enzyme activities are potential values, which do not indicate the *in situ* rates of enzymatically catalysed reactions and are not representative enough of a biogeochemical process that involves numerous enzymes (Nannipieri et al., 2018).

Surprisingly, in contrast to rhizosphere CBH activity, CBH extents were greater in spruce than in beech rhizosphere. We found a positive relationship between CBH activity extents and root hair lengths that benefited spruce, which have longer root hairs than beech. A similar association was also apparent in herbaceous plants (Ma et al., 2018). We explain this pattern by CBH particularity of being released by microorganisms, whose activities are increased by the potential rhizosphere enrichment in exudates produced by root hairs (Czarnota et al., 2003; Datta et al., 2011). Nevertheless, the relationship between the rhizosphere enzyme extent and root hair length was apparent for CBH but not for LAP and AP activities. This finding emphasises the presence of high amounts of polysaccharides, which may occur in the root hair-build rhizosheath (York et al., 2016) or may result from the fast root hair turnover (Tester and Leigh, 2001). In beech, the decrease in CBH activity resulted in a reduction of C/N acquisition ratio, which may indicate a potential increase in N mineralisation, improving soil N availability (but see Mori (2020) for the relevance of enzymatic stoichiometry). This proposition is also based on the observation that although no differences in LAP extents were apparent between beech and spruce, we may emphasise a higher ability of spruce than beech to release LAP. We observed that the Hill curve’s shape showed an abrupt decline in LAP activity compared with AP and CBH activities. In beech, the decline was in the proximity of the root centre while in spruce at the edge of the root radius. Greenfield et al. (2020) have shown that plant-release LAP activity occurs on the root surface but not in the rhizosphere, where microbial LAP activity is mainly present. We may link the observed LAP activity decline to the moment when the plant-released LAP activity ceased.

This study shows that spatial distribution of the rhizosphere enzyme activities differs between European beech and Norway spruce young seedlings which may result in different abilities to acquire nutrients and cope with nutrient limitations. The differences were apparent both in the form and strength of the relationship between the rhizoplane enzyme activities and also with regard to the extent of enzyme activities in the rhizosphere. Spruce seedlings showed higher variability in the spatial distribution of enzyme activity in the rhizoplane and rhizosphere soil than beech seedlings. In contrast, beech seedlings showed a larger positive association of enzyme activities with root morphology. We speculate that these abilities enable spruce to mobilise nutrients from heterogeneous forest soils better than beech, which in compensation, has higher plasticity to adjust biomass partitioning and root morphology and enhance enzyme activities. Beech is more successful in natural regeneration than spruce (Pretzsch et al., 2015). Spruce regeneration demands favourable light and climatic conditions to overcome the seedling establishment (Diaci, 2002; Hanssen et al., 2003; Mottet et al., 2021). However, under climate change, the strategy of spruce to mobilise nutrients by investing less in root biomass and more in enzyme distribution might surpass the beech strategy.

## Data availability statement

The raw data supporting the conclusions of this article will be made available by the authors, without undue reservation.

## Author contributions

BS and RP conceived and designed the study, analysed data, and drafted the manuscript; BR interpreted the results, and revised the manuscript and data presentations. All authors contributed to the article and approved the submitted version.

## Funding

We also acknowledge financial support from the China Scholarship Council (CSC) through a PhD scholarship to BS (No. 201506300046) and Deutsche Forschungsgemeinschaft (DFG) Priority Program 1374 “Infrastructure-Biodiversity-Exploratories” (PE 2256/3-1).

## Acknowledgments

We thank Huanying Feng for lab help and Huili Shi for support with the sampling. We are grateful to Andrea Polle for her continuous support and Mark Tibbett for the critical reading of the manuscript.

## Conflict of interest

The authors declare that the research was conducted in the absence of any commercial or financial relationships that could be construed as a potential conflict of interest.

## Publisher’s note

All claims expressed in this article are solely those of the authors and do not necessarily represent those of their affiliated organizations, or those of the publisher, the editors and the reviewers. Any product that may be evaluated in this article, or claim that may be made by its manufacturer, is not guaranteed or endorsed by the publisher.

## Supplementary material

Supplementary Figure 1 Distribution of enzyme activities on the rhizoplane of European beech. The curves present the power-law fitting of enzyme activities as a function of vertical distance from the root apex in the regions with different root hair length (RHL). Cellobiohydrolase (CBH); leucine-aminopeptidase (LAP); acid phosphomonoesterase (AP). RHL = 0 mm **(A–C)**; RHL = 0.03 mm **(D–F)**; RHL = 0.04 mm **(G–I)**; RHL > 0.04 mm **(J–L)**. The models include data from 85 individual measuring points per plant. (N = 4).

Supplementary Figure 2 Distribution of enzyme activities on the rhizoplane of Norway spruce. The curves present the power-law fitting of enzyme activities as a function of vertical distance from the root apex in the regions of different root diameter (RD). Cellobiohydrolase (CBH); leucine-aminopeptidase (LAP); acid phosphomonoesterase (AP). RD ≤ 0.24 mm **(A–C)**; RD = 0.25 mm **(D–F)**; RD = 0.26 mm **(G, H, I)**; RD = 0.27 mm **(J–L)**; RD ≥ 0.28 mm **(M–O)**.
