# Supplementary figures and images for: Contrasting distribution of enzyme activities in the rhizosphere of European beech and Norway spruce
Source: Front Plant Sci. 2022 Nov 16;13:987112. doi: 10.3389/fpls.2022.987112 (PMC9709443; doi:10.3389/fpls.2022.987112)

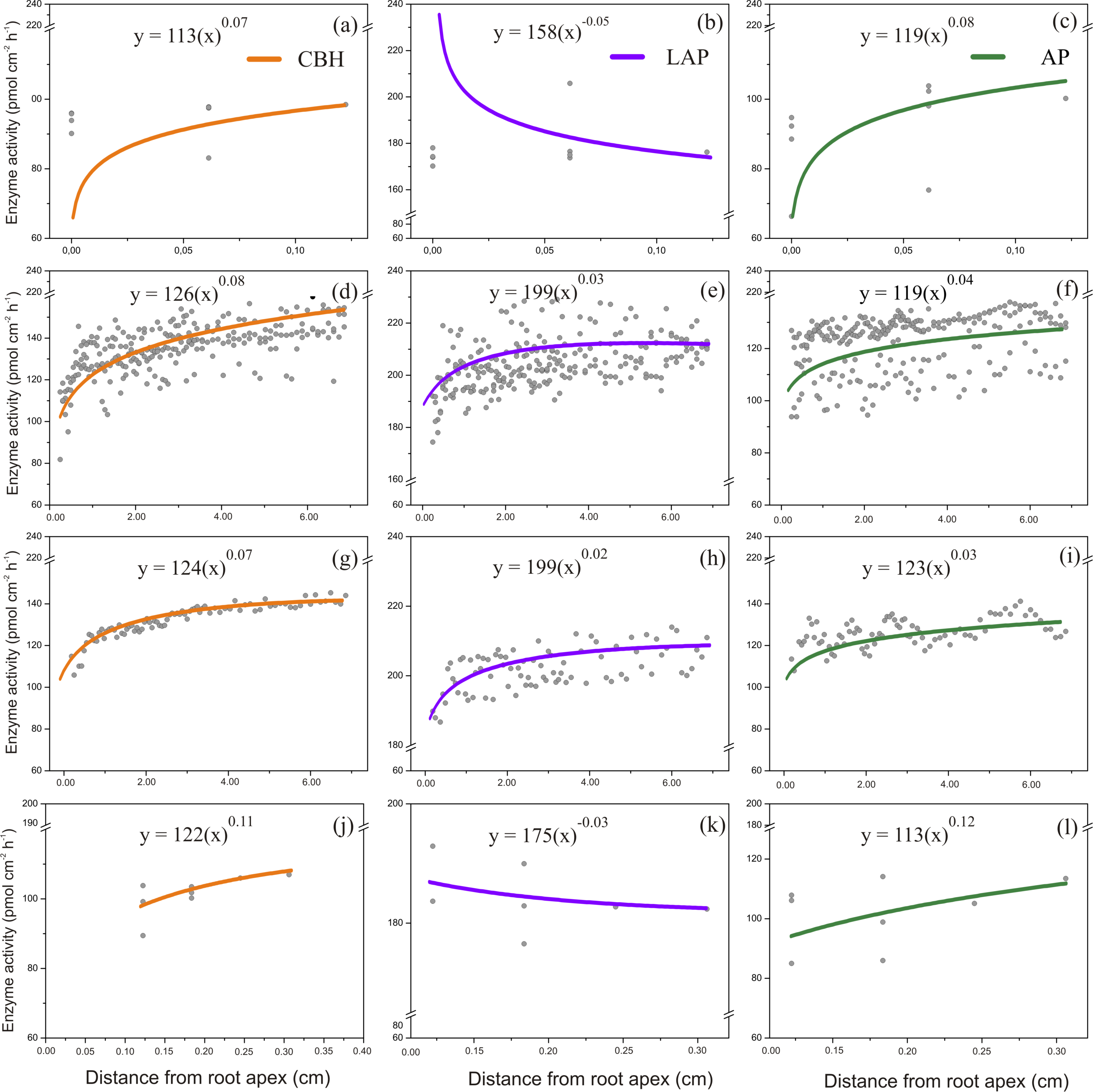

Supplement: Supplementary Figure 1 — Distribution of enzyme activities on the rhizoplane of European beech. The curves present the power-law fitting of enzyme activities as a function of vertical distance from the root apex in the regions with different root hair length (RHL). Cellobiohydrolase (CBH); leucine-aminopeptidase (LAP); acid phosphomonoesterase (AP). RHL = 0 mm (A–C); RHL = 0.03 mm (D–F); RHL = 0.04 mm (G–I); RHL > 0.04 mm (J–L). The models include data from 85 individual measuring points per plant. (N = 4). [file Image_1.tif]

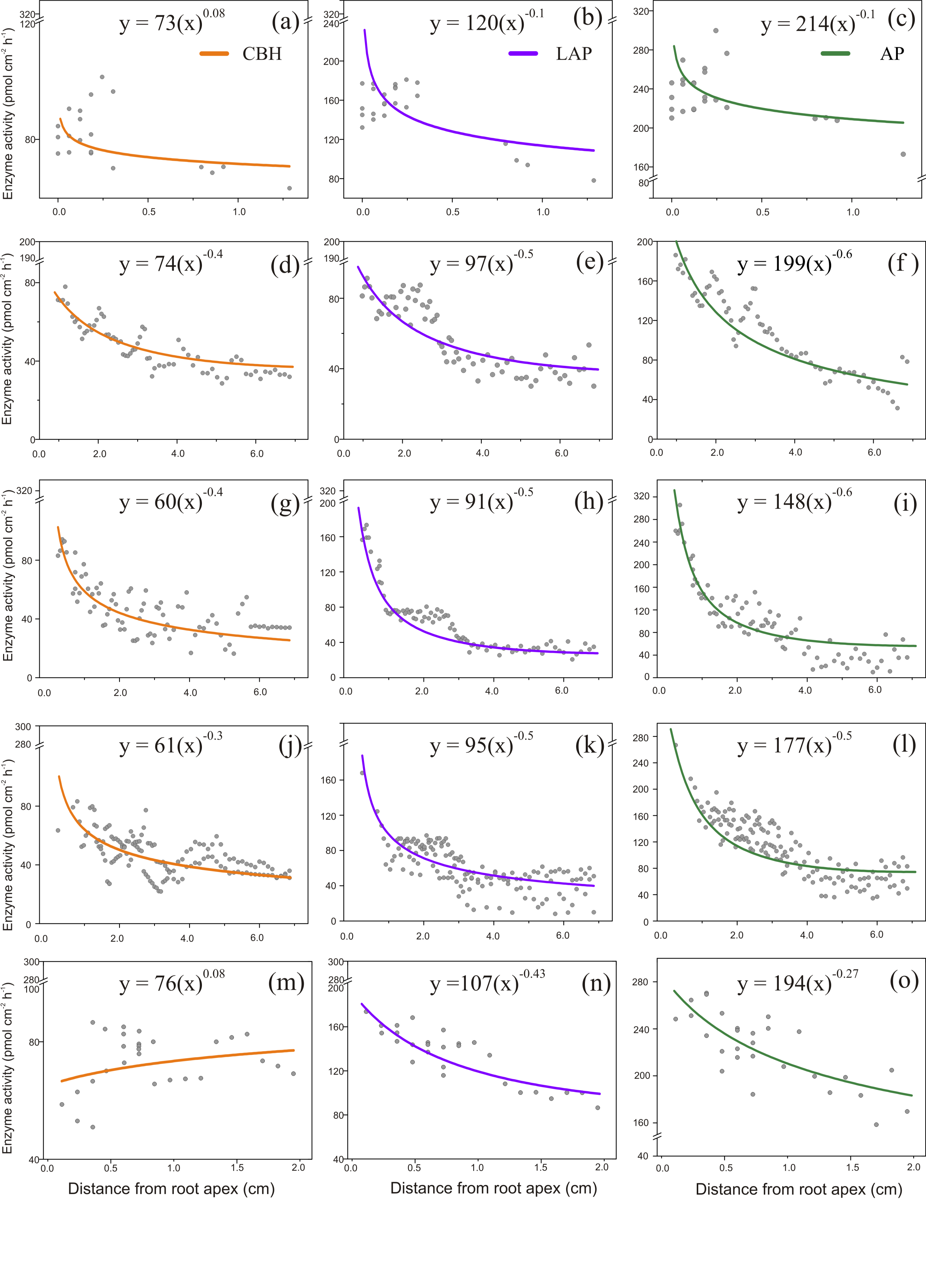

Supplement: Supplementary Figure 2 — Distribution of enzyme activities on the rhizoplane of Norway spruce. The curves present the power-law fitting of enzyme activities as a function of vertical distance from the root apex in the regions of different root diameter (RD). Cellobiohydrolase (CBH); leucine-aminopeptidase (LAP); acid phosphomonoesterase (AP). RD ≤ 0.24 mm (A–C); RD = 0.25 mm (D–F); RD = 0.26 mm (G, H, I); RD = 0.27 mm (J–L); RD ≥ 0.28 mm (M–O). [file Image_2.tif]
