# Supplementary material for: Contrasting distribution of enzyme activities in the rhizosphere of European beech and Norway spruce
Source: Front Plant Sci. 2022 Nov 16;13:987112. doi: 10.3389/fpls.2022.987112 (PMC9709443; doi:10.3389/fpls.2022.987112)
Supplement: Supplementary file 3 [file DataSheet_1.docx]

**Supplemental Table S1** Nutrition acquisition ratios C/N = ln (CBH) / ln (LAP), N/P = ln (LAP) / ln (AP), and C/P = ln (CBH) / ln (AP) and total enzyme activities = (CBH) * (LAP) * (AP) in beech and spruce rhizosphere. CBH (cellobiohydrolase), LAP (leucine-aminopeptidase), and AP (acid phosphomonoesterase). Asterisks indicate significant differences between beech and spruce (P < 0.001). N = 4.

|  | C/N | N/P | C/P | Total enzyme activity |
| --- | --- | --- | --- | --- |
| Beech | 1.08 ± 0.08 | 0.96 ± 0.14* | 1.03 ± 0.21* | 540973 ± 167* |
| Spruce | 1.04 ± 0.05 | 0.79 ± 0.11 | 0.83 ± 0.09 | 181441 ± 42 |

**Supplemental Table S2** Eigenvectors and eigenvalues of principal component analysis. N=4.

|  | Beech | | Spruce | |
| --- | --- | --- | --- | --- |
| Variable | PC1 | PC2 | PC1 | PC2 |
| Total length | 0.23 | -0.43 | -0.33 | 0.15 |
| Taproot length | 0.10 | -0.02 | -0.34 | -0.01 |
| Surface area | 0.03 | 0.27 | -0.34 | 0.05 |
| Mean Diameter | 0.35 | 0.10 | -0.14 | -0.49 |
| Volume | 0.30 | 0.24 | -0.32 | -0.16 |
| Tips | 0.16 | -0.39 | -0.33 | 0.15 |
| Forks | 0.25 | 0.35 | -0.28 | 0.34 |
| Root hair length | 0.27 | -0.35 | -0.26 | -0.24 |
| Biomass | 0.19 | 0.46 | -0.33 | -0.01 |
| Cellobiohydrolase (CBH) | 0.37 | 0.05 | 0.06 | -0.13 |
| Leucine-aminopeptidase (LAP) | 0.37 | 0.04 | -0.25 | -0.37 |
| Acid phosphomonoesterase (AP) | 0.36 | 0.11 | 0.01 | -0.58 |
